# Remote Patient Monitoring Use Among Commercially Insured Adults With Cancer

**DOI:** 10.2196/84788

**Published:** 2026-02-12

**Authors:** Joseph H Joo, Changchuan Jiang, Jessica I Billig, Nadia Lieu, Shining Yang, Yujia Jin, Arthur S Hong, Joshua M Liao

**Affiliations:** 1 Department of Internal Medicine University of Texas Southwestern Medical Center Dallas, TX United States; 2 Program on Policy Evaluation and Learning Dallas, TX United States; 3 Department of Health Economics, Systems, and Policy University of Texas Southwestern Medical Center Dallas, TX United States; 4 Department of Plastic Surgery University of Texas Southwestern Medical Center Dallas, TX United States

**Keywords:** remote patient monitoring, oncology, cancer, commercially insured, utilization

## Abstract

Our study describes the characteristics of remote patient monitoring use among commercially insured patients with cancer from 2019 to 2023.

## Introduction

Remote patient monitoring (RPM) enables continuous monitoring of vital signs, including temperature, heart rate, and blood pressure. By capturing this information while patients are at home or in their communities, RPM can detect early disease status changes and treatment-related adverse events and potentially improve outcomes through early intervention [1]. As such, RPM holds substantial promise for cancer treatment. In particular, RPM can support timely interventions to facilitate earlier recognition of decompensation, including fever and hypoxia. In the United States, however, outside of several single-centered trials, data on RPM use among patients with cancer are very limited in oncology [2,3]. Therefore, the aim of our nationwide study was to describe the characteristics of RPM use among commercially insured patients with cancer.

## Methods

We used 2019-2023 Merative MarketScan commercial claims, which contain longitudinal commercial insurance claims in the United States. The claims included nearly 40 million unique patients in the outpatient setting for the study period. We used Current Procedural Terminology codes (99091, 99453, 99454, 99457, 99458) to identify RPM services and International Classification of Diseases, Tenth Revision, Clinical Modification codes to identify patients with cancer. Cancer types were classified per American Cancer Society classifications and stratified by the cancer status (active vs history of cancer) [4]. Patients were included if they received RPM and were enrolled in insurance at least 11 out of 12 months in a calendar year.

We described the number and characteristics of patients with cancer with respect to age, sex, region, clinical complexity (ie, Charlson Comorbidity Index) and insurance plan type (health maintenance organization vs preferred provider organization vs other) [5]. We also assessed the total number of months of RPM use and the average number of months of RPM use per patient.

Because of deidentified claims data, the study was determined to be nonhuman research and therefore exempt from institutional review board at the University of Texas Southwestern Medical Center.

## Results

Between 2019 and 2023, RPM was used on 236 patients (Table 1). Half of our sample were 55-64 years old (118/236, 50.0%) and located in the southern region of the United States (117/236, 49.6%), with considerable clinical complexity (106/236, 44.9% with a Charlson Comorbidity Index of 4+). Over half of patients receiving RPM were female (141/236, 59.7%). The plurality of patients was enrolled in a preferred provider organization (89/236, 37.7%), followed by high-deductible health (64/236, 27.1%) and health maintenance organization (39/236, 16.5%) plans.

Among the 236 patients, 86.9% (205/236) used RPM for one year, while 13.1% (31/236) used RPM for 2 or more years. These patients accrued a total of 611 total months of RPM, equating to approximately 2.6 months of RPM use per patient. Of all the RPM use in patients with cancer, 45.7% (448/981) of RPM use was from those with other cancers (ie, cancer of unknown primary, rare cancers, cancer subtypes, and pre-cancers), followed by patients with cancers of the blood and lymph system (122/981, 12.4%) and patients with breast cancer (121/981, 12.3%). RPM was also used among patients with digestive (79/981, 8.1%), reproductive (77/981, 7.9%), urinary (46/981, 4.7%), endocrine (36/981, 3.7%), lung and chest (30/981, 3.1%), and skin cancers (15/981, 1.5%). RPM was least frequently used among patients with bone and soft tissue cancer (1/981, 0.1%), brain and nervous system cancer (3/981, 0.3%), and head and neck cancer (3/981, 0.3%)

**Table 1:** Characteristics of the patients with cancer using remote patient monitoring (RPM).

		Patients using RPM in 2019-2023 (N=236), n (%)	Patients using RPM for 1 year (n=205), n (%)	Patients using RPM for 2 or more years (n=31), n (%)
**Age (y)**				
	18-34	21 (8.9)	21 (10.2)	0 (0)
	35-44	32 (13.6)	28 (13.7)	4 (12.9)
	45-54	64 (27.1)	56 (27.3)	8 (25.8)
	55-64	118 (50)	99 (48.3)	19 (61.3)
	>64	1 (0.4)	1 (0.5)	0 (0)
**Sex**				
	Female	141 (59.7)	115 (56.1)	26 (83.9)
	Male	95 (40.3)	90 (43.9)	5 (16.1)
**Region**				
	South	117 (49.6)	101 (49.3)	16 (51.6)
	West	45 (19.1)	41 (20)	4 (12.9)
	North Central	35 (14.8)	34 (16.6)	1 (3.2)
	Northeast	39 (16.5)	29 (14.1)	10 (32.3)
**Insurance**				
	High deductible	64 (27.1)	59 (28.8)	5 (16.1)
	Health maintenance organization	39 (16.5)	33 (16.1)	6 (19.4)
	Preferred provider organization	89 (37.7)	77 (37.6)	12 (38.7)
	Other^a^	38 (16.1)	31 (15.1)	7 (22.6)
	Missing	6 (2.5)	5 (2.4)	1 (3.2)
**Charlson Comorbidity Index score**				
	0-1	60 (25.4)	50 (24.4)	10 (32.3)
	2-3	70 (29.7)	62 (30.2)	8 (25.8)
	4+	106 (44.9)	93 (45.4)	13 (41.9)

^a^Other insurance includes basic/major medical plan, comprehensive plan, exclusive provider organization plan, noncapitated (non-cap) point-of-service plan, and capitated (cap) or partially capitated (part cap) point-of-service plan.

## Discussion

In this nationwide US study, RPM use was limited to less than 1% (236/6,437,829) of patients with cancer and focused on particular patient groups. These exploratory findings offer several implications for future work.

First, our finding that RPM in the cancer setting was used more in older female patients reflects findings from prior noncancer studies that also showed female patients between the ages of 45 and 64 years to engage most with their clinicians through RPM [6,7]. Future research should evaluate facilitators and barriers to RPM use among younger patients with cancer, particularly given increases in early-onset cancer in the United States and likely greater familiarity with digital interventions such as RPM. Further work is needed to better understand why nearly half of the patients with RPM use were in the south region.

Second, our findings reflect uneven RPM use across cancer types, with disproportionately higher use among other cancers group (cancers of unknown primary, rare cancers, cancer subtypes, and pre-cancers) over other types such as blood and lymph system cancer. Considering that urinary, breast, lung, and digestive cancers are the most common cancer diagnoses in the United States, more work is needed to evaluate the appropriateness and impact of such findings [8]. Because not only different types of cancer but also the stages of treatment (eg, in remission, undergoing chemotherapy/radiation, postsurgery) could largely impact the indications for RPM use, future research must inform clinicians and policymakers which patients with cancer could most benefit from RPM.

Third, our finding that a patient used RPM for an average of 2.6 months indicates that RPM use is still nascent in the cancer setting. In contrast to more RPM use in chronic diseases such as hypertension (with one studying showing patients using RPM about 9 months), there is a dearth of evidence for longitudinal RPM use in cancer [9,10]. Future research is needed to evaluate the outcomes associated with RPM use to better motivate implementation among health systems.

Study limitations included descriptive design and inability to evaluate the associations between RPM use with cancer outcomes. Although RPM use in cancer could have been more prevalent through clinical trials, which are not captured in our study, the goal of our analysis was to assess real-world adoption within commercial claims data. Furthermore, our study does not capture adoption in patients with public health insurances. Ultimately, our results provide early evidence of opportunity to increase RPM use and potentially fruitful areas for future research and implementation.

## Abbreviations

RPM: remote patient monitoring

## Data Availability

The datasets generated or analyzed during this study are not publicly available due to data use and licensing restrictions from Merative MarketScan Commercial Database but are available from the corresponding author on reasonable request. The dataset analyzed during this study is the Merative MarketScan Commercial Database, which is one of the largest repositories of commercial claims data available. MarketScan is a registered trademark of Merative Corporation in the United States.
